# Protecting nursery areas without fisheries management is not enough to conserve the most endangered parrotfish of the Atlantic Ocean

**DOI:** 10.1038/s41598-020-76207-x

**Published:** 2020-11-12

**Authors:** Natalia C. Roos, Guilherme O. Longo, Maria Grazia Pennino, Ronaldo B. Francini-Filho, Adriana R. Carvalho

**Affiliations:** 1 Marine Ecology Laboratory, Department of Oceanography and Limnology, Federal University of Rio Grande do Norte, Natal, RN 59014-002 Brazil; 2 Fishing Ecology, Management and Economics Group, Department of Ecology, Federal University of Rio Grande do Norte, Natal, RN 59098-970 Brazil; 3 Spanish Institute of Oceanography, Oceanographic Centre of Vigo, 36390 Vigo, PO Spain; 4 Benthic Ecology Laboratory, Marine Biology Center (CEBIMar), University of São Paulo, São Sebastião, SP 11612-109 Brazil

**Keywords:** Ecology, Biodiversity, Conservation biology

## Abstract

Marine protected areas (MPAs) are a primary strategy for marine conservation worldwide, having as a common goal the protection of essential habitats to enhance fish population recovery. However, MPAs alone may not be effective because species are not isolated from critical impacts occurring outside their boundaries. We evaluated how protecting critical nursery habitats affect the population of an important fishing target, using a 6-year database to predict juvenile hotspots and estimate population trends of the endemic and endangered parrotfish *Scarus trispinosus* within a mosaic of MPAs at the Abrolhos Bank, NE Brazil. We found that important nursery habitats are within no-take areas, but both juvenile and adult populations still show a declining trend over time. MPAs failed to ensure population maintenance and recovery likely due to overfishing in adjacent areas and the lack of compliance to management rules within multiple-use and within no-take MPAs. MPAs alone are not enough to protect ecologically important endangered species, but is still one of the only conservation strategies, particularly in developing countries. Our results shed light on the need for a wider adoption of more effective conservation policies in addition to MPAs, both in Brazil and in countries with similar governance contexts.

## Introduction

Marine protected areas (MPAs) are one of the main management strategies to conserve and restore marine biodiversity, being especially important for vulnerable species that are under high fishing pressure^1^. The conservation of fishing-target species may be improved by the protection of key habitats for the species life cycles^2^. The identification of nursery grounds, for instance, is essential because these habitats contribute disproportionately more to the production of individuals that recruit to the adult population in comparison to other habitats used by the species during its life cycle^2^. The protection of nursery grounds and other essential habitats may be achieved through the establishment of MPAs, particularly no-take reserves^3,4^.

Key factors to consider in MPA design are the maintenance of source-sink population dynamics and connectivity among habitats used by juvenile and adult fishes^5^. Well-designed and effectively managed networks of MPAs that include source habitats may be effective not only for species conservation, but also to boost fishery yield through the spillover of fish biomass^6^. However, MPAs are often created through top-down approaches, not accounting for the participation of all stakeholders and in most cases ignoring scientific advice on optimal location and size^7^. This can be particularly problematic in developing countries where MPA implementation is often opportunistic, not accounting for socioeconomic context^8–10^. Consequently, MPAs often become ineffective due to the lack of protection for essential habitats and vulnerable species^11^.

A common way to evaluate the effectiveness of MPAs is to compare the abundance and biomass of predatory fish (such as sharks, groupers and jacks) and key herbivorous fish species inside and outside MPAs^12,13^. While large carnivores and top predators are critically important due to cascading effects^14^, key herbivorous fish such as parrotfishes (Labridae: Scarinae) perform important ecological roles on reefs, including carbonate bioerosion and grazing of algae^15,16^. Fishing pressure on this group has significantly increased in the last few decades following the decline in most predatory fish previously targeted^17,18^, causing the decline of several parrotfishes’ populations worldwide, including in the southwestern Atlantic^19^.

In Brazil, some parrotfish species have shown signs of depletion, mainly *Scarus trispinosus*, the largest southwestern Atlantic parrotfish endemic to Brazil^19^. The species has been intensively targeted in northeast Brazil^20–22^ and was considered ecologically extinct in southern Brazil^19^. Currently, *S. trispinosus* is listed as *Endangered* by the International Union for Conservation of Nature^23^ and by the Brazilian Red List of Endangered Species/BRL-EndS (Decree nº 445, Brazil's Red List, 2014), and was considered one of the most endangered parrotfishes in the world^24^. Since the early 2000’s, the species became one of the most exploited species in the Abrolhos Bank, which comprises one of the largest marine protected areas in Brazil and one of the largest remnant populations of *S. trispinosus*^12^.

Despite the implementation of MPAs, many parrotfish populations that are fishing targets often continue to decline^25^, indicating that MPAs alone are not always enough to protect parrotfishes without proper fisheries management outside the MPA’s boundaries. Another important tool to evaluate the effectiveness of MPAs and species extinction risk is to assess trends in population sizes and dynamics across areas with different restrictions to fisheries, particularly for fishing-target species that have experienced population declines^26^. We evaluated the role of a MPA network to protect the population of an endangered fish species, that remains a fishing target nevertheless. By using a 6-year database (2003–2008) on the abundance of *S. trispinosus* in the Abrolhos Bank (including no-take and multiple-use reserves), we identified spatial hotspots for juveniles (nursery areas) and estimated population trends of adults and juveniles. Our results indicate that fishing impacts in areas adjacent to MPAs may compromise their effectiveness and lead to regional declines on population sizes.

## Material and methods

### Study area

**Figure 1:**
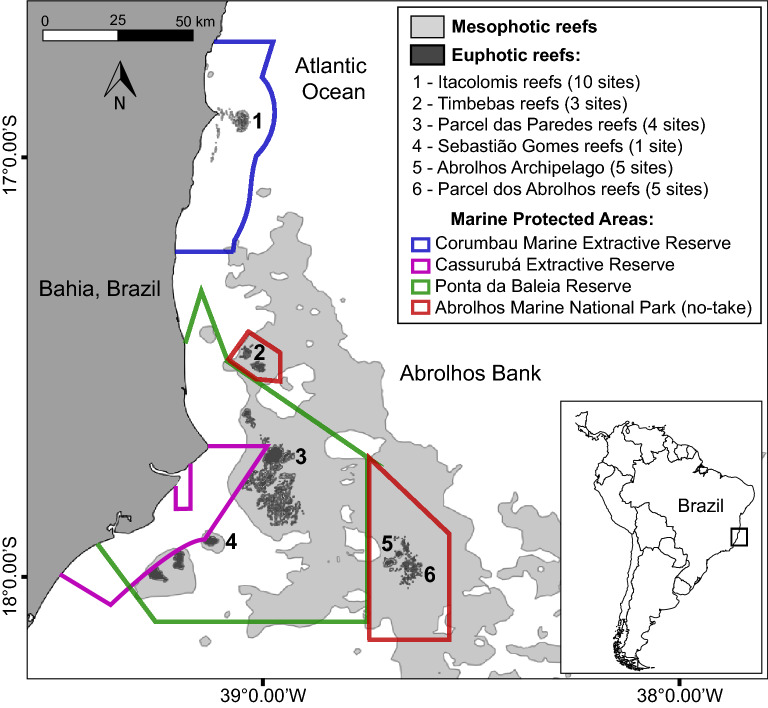
The Abrolhos Bank and Marine Protected Areas, with indication of studied reefs and number of sampling sites. The map was plotted using the packages “raster”^40^, “maptools”^41^ and “rworldmap”^42^ in the R software^31^.

We sampled 28 reefs including no-take and multiple-use reserves with depths varying from 0.5 to 27 m between 2003 and 2008 in the Abrolhos Bank, eastern Brazil (16° 40′–19° 40′ S, 39° 10′–37° 20′ W; Fig. 1). The region consists of a wide enlargement of the continental shelf (46,000 km^2^) that shelters the largest and richest reef environment in the South Atlantic^27,28^. Four different types of Marine Protected Areas (MPAs) have been established in the region, covering an area of about 6250 km^2^: Corumbau Marine Extractive Reserve, a co-managed MPA with multiple-uses created in 2000, which comprises Itacolomis reefs (1); Cassurubá Extractive Reserve, a co-managed MPA with multiple-uses created in 2009; Ponta da Baleia Reserve, a large multiple-use MPA created in 1993, which comprises Parcel das Paredes (3) and Sebastião Gomes reefs (4), and is considered as a “*Paper Park*” due its lack of proper management^28^; and the Abrolhos Marine National Park, a no-take MPA crated in 1983, which comprises two distinct portions, one inshore and poorly enforced (Timbebas reefs [2]) and another offshore and more intensively enforced (Abrolhos Archipelago [5] and Parcel dos Abrolhos reefs [6]; Fig. 1).

### Fish and benthic surveys

In each of the 28 sites, juveniles (i.e. individuals ≤ 10 cm of total length, TL, around 4 months old^22^) and adults (i.e. individuals > 10 cm of TL) of *S. trispinosus* were counted through a nested stationary visual census technique^29^. The density of *S. trispinosus* in each visual census was calculated by the equation N/π × *r*^2^, where N is the number of individuals counted and π × *r*^2^ is the sampling area, which *r* = 2 (12.6 m^2^) for juveniles and *r* = 4 (50.2 m^2^) for adults. Field protocols were approved by the Brazilian legislation (ICMBio-MMA—Brazilian Ministry of Environment) and sampling was carried out using observational and non-destructive techniques, under the permit SISBIO-11709-1. The fish surveys were conducted in the inshore reefs between 2003 and 2008, and in the offshore reefs between 2005 and 2008, totalling 3987 visual censuses (details in Supplementary Table S1). All surveys were conducted during the austral summer (January–April). Benthic cover data was obtained from Francini-Filho et al*.*^27^ and used as predictors for juveniles’ distribution. Seven morpho-functional groups of benthic organisms were used: turf algae (i.e. epilithic algal matrix), crustose calcareous algae (CCA), fire corals (milleporids), fleshy macroalgae, sponges, stony corals (scleractinians) and zoanthids.

### Data analysis

#### Density of juveniles and adults

We assessed differences in the density of juveniles and adults among areas using permutation-based analysis of variance (Permutation-based ANOVA), which does not require normality or homogeneity of variances, using the package “lmperm”^30^ (aovp function) in R software^31^. We ran a post hoc pairwise permutational test to assess the significant contrasts, using the package “rcompanion”^32^ (pairwise PermutationTest function). In order to calculate mean densities per location, we aggregated the abundance data from all visual censuses with all years combined (n = 3987; mean densities = N individuals/N visual censuses per location). Mean densities were plotted using the package “yarrr”^33^ (pirateplot function).

#### Modelling juveniles’ distribution

In order to identify the hotspots of *S. trispinosus* juveniles, we used hierarchical Bayesian hurdle spatio-temporal models. These types of models are implemented to deal with high numbers of zero in the response variable (*S. trispinosus*’ juveniles), in two stages: (1) modelling presence/absence in order to obtain the envelope of the predicted probability of presence of the species studied (binomial distribution) and (2) modelling the juveniles’ density (Gamma distribution; Shapiro and Kolmogorov–Smirnov normality tests, p-value ≤ 0.001) of the studied species only in areas where species were predicted to be present^34^. For both stages, the explanatory variables included all environmental and benthic variables, the observer random effect, the year factor and a spatially structured random effect that account for the spatial autocorrelation. The models were performed using the Integrated Nested Laplace Approximations (INLA) approach^35^ and the package implemented in R software^31^. For fixed parameters, vague priors were assigned with zero mean and a variance of 100.

Variable selection was performed beginning with all possible interaction terms, but only the best combination of variables was chosen. Such choice was based on three different measures: (1) the Watanabe-Akaike information criterion (WAIC), (2) the Root Mean Square Error (RMSE), and (3) the adjusted coefficient of determination (R^2^). The best (and most parsimonious) model was chosen based on the compromise between low WAIC values, low RMSE values, and high R^2^ values, containing only relevant predictors, i.e., those predictors with 95% confidence intervals not covering zero. Functional responses were plotted using “ggplot2” package^36^ in R software. In addition to the benthic cover, five environmental variables were also considered as potential predictors of juveniles’ distribution^37^: Sea Surface Temperature (SST in °C) and Sea Surface Salinity (SSS in PSU), depth (in meters), rugosity (low = 0 vs high = 1) and distance to land (in meters; details in Supplementary Methods: Environmental variables).

Predictions were obtained for each year from 2003 to 2008 using the annual mean of the selected environmental and benthic variables. In addition, an average map of the entire period was also generated using the environmental means of the entire time series as predictors (see details of the temporal variation of SST and SSS in Supplementary Figs. S1, S2). Prediction validation was performed using two separated approaches. Firstly, the predicted and observed values using the full dataset were compared. Secondly, a tenfold cross validation using a random half of the dataset was performed to build the model and the remaining data to test the prediction. In both cases two statistics were calculated: Pearson’s correlation coefficient r and the average error (AVEerror^38^).

### Modelling abundance trends

Abundance trends of both juveniles and adults were modelled using Bayesian time series models. Sites were aggregated within each location, and adults’ and juveniles’ abundance trends were modelled separately for each location, as well as for the entire region.

Three different models were used in each case: (1) an autoregressive model of order 1 (AR1), (2) a random walk model of order 1 (RW1) and (3) a random walk model of order 2 (RW2)^39^. For all models a Poisson distribution was implemented and the linear predictor was linked to the mean using the natural logarithm. Comparison among models were performed using the Watanabe-Akaike information criterion (WAIC) and Conditional Predictive Ordinates (CPO). While WAIC values indicate the goodness of fit of the models, the CPO evaluates the predictive capacity. The best (and most parsimonious) model was chosen based on the compromise between low WAIC and CPO values. All models were performed using the Integrated Nested Laplace Approximations (INLA) approach and the package^35^ implemented in R software.

## Results

### Density of juveniles and adults

**Figure 2:**
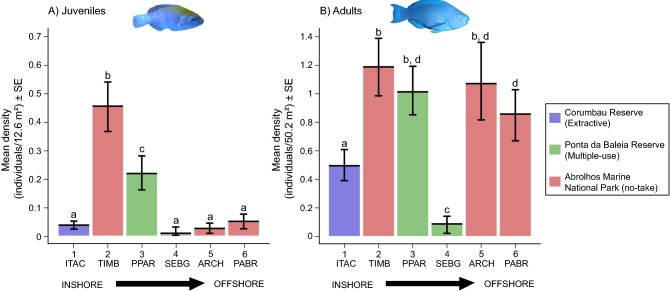
Density of *Scarus trispinosus*’ (**A**) juveniles and (**B**) adults in the Abrolhos Bank reefs. *ITAC* Itacolomis reefs (1); *TIMB* Timbebas reefs (2); *PPAR* Parcel das Paredes reefs (3); *SEBG* Sebastião Gomes reefs (4); *ARCH* Abrolhos Archipelago (5); *PABR* Parcel dos Abrolhos reefs (6). Different colors indicate different Marine Protected Areas. Different letters indicate significant differences at a 5% significance level. Permutation-based ANOVA, Degrees of Freedom = 5, Iter = 5000, p-value = 0.001, (**A**) Juveniles—sum of squares = 87.13, mean sum of squares = 17.426; (**B**) Adults—sum of squares = 66.17, mean sum of squares = 13.23. *SE* standard error. Please note that graphs within this plate are in different scales to accommodate and represent variation between juveniles’ and adults’ densities. Graphs were plotted using the package “yarrr”^33^ in the R software^31^.

Mean densities differed significantly among locations, both for juveniles and adults (Permutation-based ANOVA, p < 0.05). The inshore reefs of Timbebas (2) sustained the highest density of juveniles (~ 0.45 ind./12.6 m^2^, pairwise permutation test, p < 0.05), followed by Parcel das Paredes (3; ~ 0.2 ind./12.6 m^2^, pairwise permutation test, p < 0.05). Density of juveniles did not vary among the rest of four areas (pairwise permutation test, p > 0.05, Fig. 2A). Density of adults was also highest in the inshore reefs of Timbebas (2; ~ 0.45 ind./50.2 m^2^), followed by Parcel das Paredes reefs (3; ~ 0.4 ind./50.2 m^2^), and the offshore reefs of Abrolhos Archipelago (5; ~ 0.4 ind./50.2 m^2^) and Parcel dos Abrolhos reefs (6; ~ 0.35 ind./50.2 m^2^), with significant differences between Timbebas reefs (2) and Parcel dos Abrolhos (6; permutation test, p < 0.05). The lowest densities were recorded in the Itacolomis (1) and Sebastião Gomes reefs (4), with significant differences between these two locations (permutation test, p < 0.05, Fig. 2B).

### Juveniles’ distribution

The best-fitted model presented a good prediction, indicated by the high values for the Pearson’s correlation coefficient both for the original dataset (0.83, p < 0.05) and for the cross validation done with half of the dataset (0.85, p < 0.05). Likewise, low values for the AVEerror were achieved in both the original (AVEerror = 0.02) and in the cross validation (AVEerror = 0.03) datasets.

**Figure 3:**
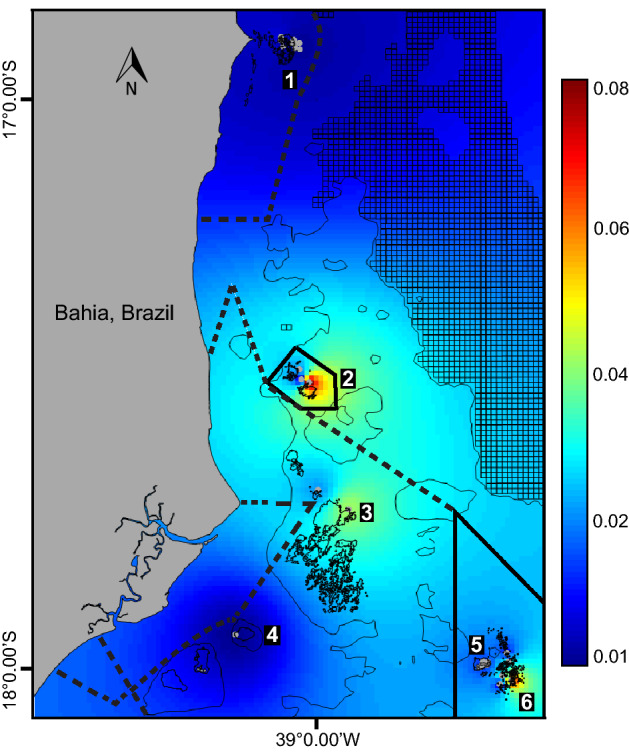
Predicted density for *Scarus trispinosus*’ juveniles (mean of 2003–2008). 1. Itacolomis reefs; 2. Timbebas reefs; 3. Parcel das Paredes reefs; 4. Sebastião Gomes reefs; 5. Abrolhos Archipelago; 6. Parcel dos Abrolhos reefs. Polygons with solid lines indicate no-take areas and with dashed lines indicate areas where fisheries are allowed under specific regulations. The map was plotted using the packages “raster”^40^, “maptools”^41^ and “rworldmap”^42^ in the R software^31^.

**Figure 4:**
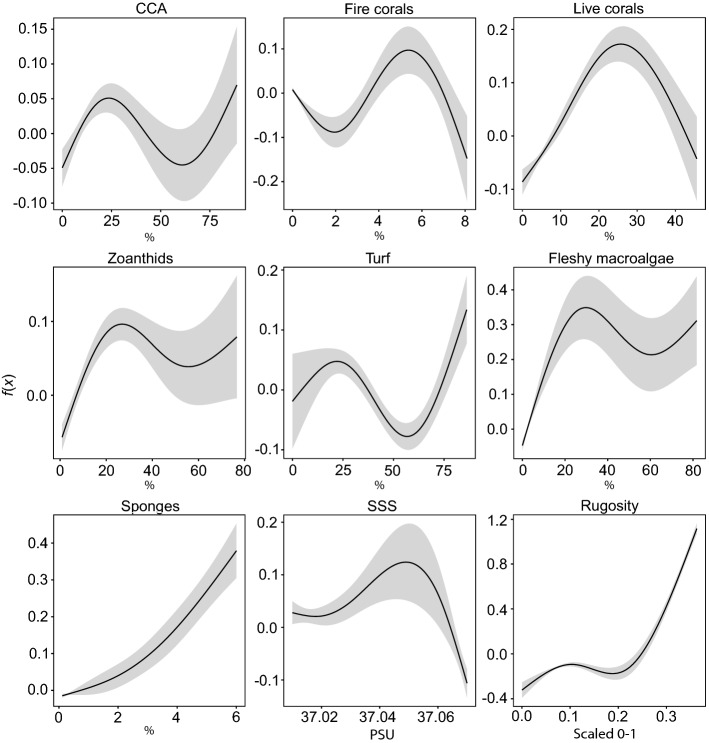
Functional response of the relevant variables selected to explain the hotspots of *Scarus trispinosus*’ juveniles (mean of 2003–2008). The solid line is the smooth function estimate and shaded regions represent 95% credibility interval (CI). *CCA* crustose calcareous algae, *SSS* sea surface salinity. Graphs were plotted using the package “ggplot2”^36^ in the R software^31^.

The predicted major nurseries of *S. trispinosus* in the Abrolhos Bank were Timbebas (2), Parcel das Paredes (3) and Parcel dos Abrolhos reefs (6; Fig. 3; average predictive map 2003–2008; see details by year in Supplementary Fig. S3). Based on juveniles’ density, the best-fitted model predicted their preferred habitat requirements, which were: higher rugosity, CCA, turf, zoanthids, fleshy macroalgae and sponges cover, and a SSS optimum value around 37.05 PSU (Fig. 4). Similarly to the SSS, fire corals and stony corals also presented an optimum range of correlation with the juveniles, with optimum values around 6% of fire corals cover and 30% of stony corals cover (Fig. 4). The best-fitted model also included the year factor that account for the temporal variability and the random spatial effect that account for the spatial intrinsic variability of the data (Supplementary Table S2).

### Population’s abundance trends

**Figure 5:**
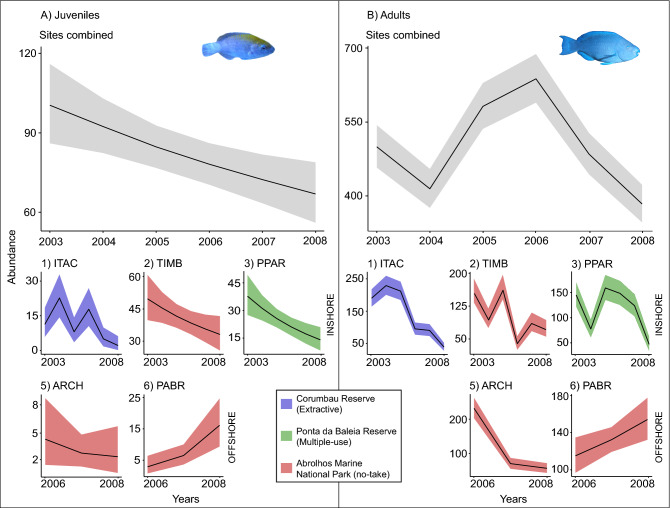
Abundance trends for *Scarus trispinosus*’ (**A**) juveniles and (**B**) adults between 2003 and 2008. The models comprise sites combined and each studied reef, except for Sebastião Gomes reefs. *ITAC* Itacolomis reefs (1); *TIMB* Timbebas reefs (2); *PPAR* Parcel das Paredes reefs (3); *ARCH* Abrolhos Archipelago (5); *PABR* Parcel dos Abrolhos reefs (6). Different colors indicate different Marine Protected Areas. The shaded regions represent 95% credibility interval (CI). Please note that graphs within this plate are in different scales to accommodate and represent the within site variation. Graphs were plotted using the package “ggplot2”^36^ in the R software^31^.

According to the best-fitted model, the total abundance of both juveniles and adults of *S. trispinosus*’ declined in the Abrolhos Bank between 2003 and 2008 (Supplementary Table S3; Fig. 5). Models per site showed these declining trends for all reefs, except for Parcel dos Abrolhos reefs (6), where abundance of both juveniles and adults increased between 2006 and 2008 (Fig. 5). It was not possible to unveil abundance trends for Sebastião Gomes reefs (4) due to its low number of juveniles (n = 1 in 2005) and adults (n = 9 between 2003 and 2008). The year 2005 was not included in the models of Abrolhos Archipelago (5) and Parcel dos Abrolhos reefs (6) due to the low number of visual census conducted during this year in these reefs (details in Supplementary Table S1).

## Discussion

Many studies show the benefits of Marine Protected Areas (MPAs) in conserving and restoring marine biodiversity^1,13,43^. Despite their critical importance, in some cases MPAs alone are not an effective strategy because species are not protected from critical impacts outside the MPAs boundaries^44^. We found that despite two out of the three most important nursery habitats for *Scarus trispinosus* being within no-take areas of a MPA (i.e. Timbebas [2] and Parcel dos Abrolhos reefs [6]; Fig. 3), both juvenile and adult populations showed declining trends over time within such areas. The declining trends suggest that the excessive removal of adult individuals from the population outside no-take MPAs and inside multiple-use reserves may be decreasing the generation of new recruits. Both results combined reinforce that, despite being essential, MPAs cannot be the only conservation tool to protect an endangered species targeted by fisheries. In such cases, specific conservation policies in addition to MPAs must be adopted.

The density of *S. trispinosus*’ juveniles was considerably higher in reefs closer to the coast (i.e. Timbebas [2] and Parcel das Paredes reefs [3]; Fig. 2A), which consist of shallow (1–18 m deep) and structurally complex reefs with a characteristic form of mushroom-shaped pinnacles^28^. Inshore shallower reefs and other coastal marine habitats are important nursery grounds for many reef fishes, because these habitats have more food resources and refuges for juveniles, and lower predation risk compared to adult habitats^2,45,46^. Due to the wide expanse of the continental shelf in the Abrolhos region, some shallow reefs occur even far from the coast^28^. This is the case of the reefs at Parcel dos Abrolhos (6), located 60 km from the coast, which are among the habitat hotspots for juveniles. Despite being slightly deeper, Parcel dos Abrolhos (6) harbours similar habitat conditions compared to the hotspots in the inshore reefs, but with a lower juvenile density, likely due to the greater distance from the coast and slightly greater depths (Fig. 2A; Fig. 3).

All benthic cover variables were important to determine the juvenile hotspots, likely because the benthic morpho-functional groups used in the analysis are abundant and well distributed in all study sites^27^ (Fig. 4). Reef attributes and the availability of preferred food sources are commonly within the main drivers of parrotfish distribution^47^. Some of the substrates selected by the best-fitted model are recognized as the main grazing targets of *S. trispinosus*, especially crustose coralline algae (CCA), turf and fleshy macroalgae^48^. In addition, fire and stony corals were important to sustain high abundance of *S. trispinosus* juveniles, indicating that the maintenance of healthy reefs and corals is critical in nursery habitats for this species. Although *S. trispinosus* may also feed on stony corals, the species allocate only a small fraction of its bites to this type of substrate^49^. Therefore, it is more likely that the associations of juveniles with fire and stony corals relate to the structural complexity provided by these invertebrates, rather than the species feeding activity.

Despite the physical and biological similarities among the three hotspots of juvenile abundance (Timbebas [2], Parcel das Paredes [3] and Parcel dos Abrolhos [6]), the enforcement is critically different among these areas. Although there was some temporal variation in the importance of the hotspots (Supplementary Figure S3), the location and protection level within each area were the same throughout the years. The offshore portion of the Abrolhos Marine National Park, that includes Parcel dos Abrolhos reefs (6), have a stronger enforcement due to the presence of the Federal Environmental Agency and the Brazilian Navy in the Abrolhos Archipelago. On the contrary, Timbebas reefs (2) are weakly enforced and poaching occurs frequently^12^. Even so, Timbebas reefs are one of the most important areas in the Abrolhos Bank in terms of abundance and biomass of small carnivores and large herbivores, including *S. trispinosus*^12^, indicating that the higher density of juveniles mainly emerged from habitat requirement and not necessarily from the MPA protection alone, since small-sized juveniles are not fishing targets. On the other hand, Parcel das Paredes reefs (3), which is the largest complex of inshore shallow reefs in the region^50^ and one of the main nurseries areas, had a significantly lower juvenile’ density compared to Timbebas (2), despite having habitat similarities (Fig. 2A). Differently from Timbebas (2), Parcel das Paredes reefs (3) are located in a multiple-use reserve subjected to constant fishing pressure due to its lack of proper management. Therefore, the low juvenile density in Parcel das Paredes (3) can also result from high fishing pressure on adults, reducing the supply of juveniles in this area.

Almost two tons of *S. trispinosus* is fished per month by artisanal fisheries in the Abrolhos Bank^51^. Moreover, the species is one of the main targets of recreational spearfishers in the region^52,53^. Overfishing may affect the demographic structure of parrotfish populations by modifying their vital rates of mortality and inducing species to change sex at smaller age and sizes^54,55^. This is especially problematic for a relatively larger, late maturing and longer-lived hermaphrodite parrotfish such as *S. trispinosus*^21,22^. MPAs may prevent some of these effects by exporting larger older individuals to surrounding areas, and/or by receiving recruits that can fully develop to adult sizes within MPA boundaries^6,56^. Well-designed networks of MPAs can maintain source-sink population dynamics and its temporal variability if important habitats such as nurseries and breeding areas are effectively protected^5^. For *S. trispinosus*, which is known to undergo an ontogenetic habitat shift from inshore to offshore reefs, with juveniles being more abundant near the coast and mature adult individuals in deeper offshore reefs^21,22^, protecting inshore and offshore habitats is essential to maintain the species source-sink population dynamics. The Abrolhos Marine National Park (no-take) seems to be a good model of that, since it comprises both inshore and offshore portions. However, when fishing pressure outside the MPA is strong enough, the input of larvae decreases as the adult population declines^57^. Intense fishing pressure on *S. trispinosus* outside the MPA and the occasional poaching occurring inside the MPAs, related to lack of compliance and proper enforcement, may explain the declining pattern of adult and juvenile populations. The protection of the main nurseries habitats is not preventing the species from declining, probably because of the stronger fishing pressure on adults, both inside multiple-use reserves and unprotected reefs. In other words, the lack of protection for adults, which could exist through fisheries management in addition to MPAs, may be compromising the species’ life cycle. Surprisingly, the offshore Parcel dos Abrolhos (6) was the only location that experienced population increases between 2006 and 2008 (Fig. 5). In addition to being a better enforced area, the population from Parcel dos Abrolhos (6) may be less susceptive to other coastal disturbances due to its larger distance from the coast, may acting as a refugium^58,59^ for *S. trispinosus*. The structural complexity of Parcel dos Abrolhos reefs (6), which is similar to Timbebas (2) and Parcel das Paredes reefs (3), may provide preferable habitat conditions for the species. These factors may explain the slight population increase in Parcel dos Abrolhos (6) while populations declined in the other locations.

Currently, no-take zones within MPAs cover only about 3% of the Abrolhos Bank. All other shallow reefs are open-access (i.e. unprotected), although some of them belong to marine extractive reserves and have some level of regulation. Recent efforts have aimed the expansion of the Abrolhos Marine National Park. The target areas of protection are deeper reefs in the east side of the Abrolhos Marine National Park. These areas aggregate high biomass of commercially important fishes, and consequently, fishing activities intensively occur in the area^60^. Although the predominant occurrence in shallower waters, evidences indicate that larger mature individuals of *S. trispinosus* also occurs in offshore reefs between 50 and 60 m deep^21,60,61^, suggesting that the expansion of the Abrolhos Marine National Park toward deeper reefs may benefit the species, specially by protecting adult individuals*.* Besides that, proper management of Ponta da Baleia Reserve and the inclusion of no-take zones in this area could benefit inshore populations of Parcel das Paredes reefs (3), which is one of the juveniles’ hotspots and remain poorly protected. Despite the encouraging expectations derived from expanding protection areas, our results suggest that expanding protected areas without setting clear rules for managing the areas, pursuing compliance among stakeholders and intensifying the enforcement inside no-take areas, may be insufficient to protect fishing-target endangered species such as *S. trispinosus.* This is the case of many regions worldwide where fishing-target species declined despite MPAs creation efforts^44,62^, including other iconic species, the bumphead parrotfish (*Bolbometopon muricatum*) in the Solomon Islands^63^.

Even though MPAs may enhance the persistence of exploited parrotfishes^13^, we showed that the protection of the main nursery areas of *S. trispinosus* is not enough to prevent the declining trend in the juvenile’s abundance, likely because of intensive fishing of adults in adjacent areas. In 2014, *S. trispinosus* fishing was nationally banned after the species was listed as endangered on the Brazilian Red List of Endangered Species (Decree No. 445), but with no significant enforcement. Most recently, in 2018, the Brazilian National Recovery Plan for endangered species (Decree No. 59-B) regulated the species fishing under restrict rules, which included the ban on recreational fishing and fishing nets, determined spearguns as the only fishing gear allowed and a slot-size limit for catches between 39 and 63 cm total length. The proposed slot-size limit aims to protect both immature and older mature individuals (including most males) which are those with a greater reproductive capacity, according to the species’ demographic traits^21,22^. The plan also proposes that fishing would only be allowed within multiple-use marine protected areas by authorized artisanal fishers, with continuous monitoring. The Abrolhos bank seems to be a suitable region to enforce these measures, due to the presence of multiple-use reserves and the fact that most of the artisanal catches are within the proposed slot size limit^21^. Until the present moment, however, the government has not enforced the plan and the species keep being fished indiscriminately. Given the critical situation, efforts to implement the management measures should be taken as soon as possible, otherwise, the complete fishing ban will be the only way to aid the species recovery.

We are aware that our estimates would benefit from a longer timeseries, but given the significant declines and increasing threats reported to this species^19,21–23^, evaluating population trends and the effectiveness of conservation measures is imperative. Therefore, in the absence of such longer term data we modelled the six-year time series using a robust method that provided reliable estimates accounting for this potential limitation. Also, one can argue that the temporal trends we observed could be due to other anthropogenic impacts rather than fisheries, such as climate change or invasive species. However, the Abrolhos bank did not experience any significant effect related to climate change or thermal anomalies^64^ and had minimal variability in sea temperature and salinity within the time span of our study (see Supplementary Fig. S1 and Fig. S2). Also, the region did not experience problems related to invasive species back in the early 2000’s. Recently, the expansion of the invasive coral species of the genus *Tubastraea* in the southern Abrolhos Bank^65^ (more than 100 km from our study sites) raised concerns, but no invasive fish species was ever recorded in the region. Therefore, the strongest and most consistent impact on *S. trispinosus* populations is fishing pressure, which removes about two tons of the species from the Abrolhos Bank monthly^51^. Conservation actions targeting this species should focus on fisheries management, enforcement and compliance to guarantee its long-term survivorship.

The creation process of new MPAs and fishery management plans must involve fishers and other stakeholders in order to reach compliance and avoid the dissemination of paper parks^66^. Parrotfish populations, including *S. tripisnosus* at the Abrolhos Bank, and other important fishing-target and endangered species elsewhere, will only benefit from MPA networks^1,43^ if it comes associated to other conservation strategies, including a well-implemented demographic-based fisheries management plan that considerably reduce fishing pressure.

## Supplementary information

Supplementary Information

## Data Availability

The data and codes are available at https://zenodo.org/record/3964327#.XyF1WZ5KgdW.
