# Author Correction: Protecting nursery areas without fisheries management is not enough to conserve the most endangered parrotfish of the Atlantic Ocean

**DOI:** 10.1038/s41598-021-85212-7

**Published:** 2021-03-08

**Authors:** Natalia C. Roos, Guilherme O. Longo, Maria Grazia Pennino, Ronaldo B. Francini‑Filho, Adriana R. Carvalho

**Affiliations:** 1 Marine Ecology Laboratory, Department of Oceanography and Limnology, Federal University of Rio Grande Do Norte, Natal, RN 59014-002 Brazil; 2 Fishing Ecology, Management and Economics Group, Department of Ecology, Federal University of Rio Grande Do Norte, Natal, RN 59098-970 Brazil; 3 Spanish Institute of Oceanography, Oceanographic Centre of Vigo, 36390 Vigo, PO Spain; 4 Benthic Ecology Laboratory, Marine Biology Center (CEBIMar), University of São Paulo, São Sebastião, SP 11612-109 Brazil

Correction to: *Scientific Reports* 10.1038/s41598-020-76207-x, published online 12 November 2020

The Author Contributions section in this Article is incorrect.

“R.B.F.F. collected the data; N.C.R. and M.G.P. analyzed the data; N.C.R., G.O.L. and A.R.C. wrote the paper.”

should read:

“R.B.F.F. conceived and designed the analysis and collected the data; N.C.R. and M.G.P. analyzed the data; N.C.R. and G.O.L. wrote the paper, with input and revision from all authors (N.C.R., G.O.L., M.G.P., R.B.F.F. and A.R.C.). We have no conflicts of interests to declare.”

