# Supplementary material for: Protecting nursery areas without fisheries management is not enough to conserve the most endangered parrotfish of the Atlantic Ocean
Source: Sci Rep. 2020 Nov 12;10:19143. doi: 10.1038/s41598-020-76207-x (PMC7661516; doi:10.1038/s41598-020-76207-x)
Supplement: Supplementary file 1 — Supplementary Information [file 41598_2020_76207_MOESM1_ESM.docx]

**Protecting nursery areas without fisheries management is not enough to conserve the most endangered parrotfish of the Atlantic Ocean**

Natalia C. Roos^1*^, Guilherme O. Longo^1^, Maria Grazia Pennino^2,3^, Ronaldo Francini-Filho^4^, Adriana R. Carvalho^2^

^1^Marine Ecology Laboratory, Department of Oceanography and Limnology, Universidade Federal do Rio Grande do Norte, Natal, RN, 59014-002, Brazil. ^2^Fishing Ecology, Management and Economics group, Department of Ecology, Universidade Federal do Rio Grande do Norte, Natal, RN, 59098-970, Brazil. ^3^Spanish Institute of Oceanography, Oceanographic Centre of Vigo, Vigo, PO, 36390, Spain. ^4^Benthic Ecology Laboratory, Marine Biology Center (CEBIMar), University of São Paulo, São Sebastião, SP, 11612-109, Brazil. *Corresponding author: nataliaroos@gmail.com.

**Material and Methods**

**Table S1.** Sample summary of the field effort between 2003 and 2008 across the 28 studied sites in the Abrolhos Bank, eastern Brazil. na = not available.

| **Reef area /**  **Sampling sites** | **Coordinates** | **N fish surveys/year** | | | | | | **Sum of samples** |
| --- | --- | --- | --- | --- | --- | --- | --- | --- |
|  |  | **2003** | **2004** | **2005** | **2006** | **2007** | **2008** |  |
| **Itacolomis reefs (1)** | |  |  |  |  |  |  |  |
| A1 | -16.899 / -39.063 | 30 | 30 | 34 | 40 | 30 | 30 | **194** |
| A2 | -16.905 / -39.041 | 29 | 30 | 30 | 30 | 30 | 30 | **179** |
| A3 | -16.903 / -39.031 | 15 | 15 | 19 | 15 | 11 | 15 | **90** |
| B1 | -16.896 / -39.063 | 30 | 30 | 36 | 30 | 30 | 29 | **185** |
| B2 | -16.901 / -39.042 | 30 | 30 | 30 | 36 | 30 | 39 | **195** |
| C1 | -16.892 / -39.063 | 47 | 48 | 35 | 34 | 30 | 14 | **208** |
| C2 | -16.898 / -39.040 | 30 | 30 | 35 | 34 | 30 | 30 | **189** |
| AMP1 | -16.909 / -39.060 | 30 | 30 | 30 | 30 | 28 | 28 | **176** |
| AMP2 | -16.912 / -39.038 | 47 | 49 | 35 | 38 | 30 | 31 | **230** |
| AMP3 | -16.914 / -39.030 | 14 | 15 | 15 | 19 | 15 | 15 | **93** |
| **Timbebas reefs (2)** | |  |  |  |  |  |  |  |
| TIM1 | -17.482 / -39.013 | 30 | 19 | 30 | 30 | 30 | 30 | **169** |
| TIM2 | -17.477 / -39.027 | 30 | 29 | 30 | 30 | 31 | 30 | **180** |
| TIM3 | -17.460 / -39.031 | 30 | 30 | 30 | 30 | 31 | 30 | **181** |
| **Parcel das Paredes reefs (3)** | |  |  |  |  |  |  |  |
| ARENG | -17.666 / -38.997 | 30 | 30 | 27 | 30 | 31 | 30 | **178** |
| PA1 | -17.699 / -38.942 | 30 | 28 | 30 | 30 | 30 | na | **148** |
| PLES | -17.783 / -39.051 | 30 | 30 | 33 | 31 | 31 | 30 | **185** |
| PSUL | -17.880 / -38.938 | 30 | 30 | 30 | 30 | 30 | 30 | **180** |
| **Sebastião Gomes (4)** |  |  |  |  |  |  |  |  |
| SG | -17.913 / -39.145 | 30 | 30 | 30 | 30 | 29 | 30 | **179** |
| **Abrolhos Archipelago (5)** | |  |  |  |  |  |  |  |
| FAROL | -17.965 / -38.694 | na | na | 30 | 34 | 15 | 15 | **94** |
| GUARITA | -17.960 / -38.692 | na | na | na | 36 | 15 | 14 | **65** |
| MVERDE | -17.964 / -38.702 | na | na | na | 33 | 14 | 15 | **62** |
| PNORTE | -17.959 / -38.701 | na | na | na | 34 | 21 | 15 | **70** |
| SIRIBA | -17.968 / -38.707 | na | na | na | 33 | 15 | 15 | **63** |
| **Parcel dos Abrolhos reefs (6)** | |  |  |  |  |  |  |  |
| PAB1 | -17.991 / -38.650 | na | na | na | 28 | 29 | 30 | **87** |
| PAB2 | -17.983 / -38.667 | na | na | na | 49 | 30 | 30 | **109** |
| PAB3 | -17.998 / -38.671 | na | na | na | 31 | 30 | 30 | **91** |
| PAB4 | -17.959 / -38.655 | na | na | 29 | 29 | 30 | 30 | **118** |
| PAB5 | -17.944 / -38.659 | na | na | na | 30 | 29 | 30 | **89** |
| **Total** |  | **542** | **532** | **568** | **714** | **655** | **621** | **3987** |

**Environmental variables**

Oceanographic variables were obtained from Copernicus Marine Environment Monitoring Service (CMEMS), which provides daily information on the physical state, variability and dynamics of the ocean and marine ecosystems worldwide. In particular, Sea Surface Temperature (SST) and Sea Surface Salinity (SSS), were extracted, with a spatial resolution of 0.25 x 0.25 degree, as monthly means and were aggregated in yearly maps using the *Spatial Analysis* tool of ArcGIS 10.

Bathymetry and distance to land were retrieved from the MARSPEC database, (http://www.marspec.org). MARSPEC is a world ocean dataset with a spatial resolution of 0.01 x 0.01 degrees developed for marine spatial ecology^1^. Conversely, rugosity was derived from the bathymetry map using the “Terrain Ruggedness (VRM)” of the “Benthic Terrain Modeler” tool in ArcGIS 10.2.2. The VRM measures rugosity as the variation in three-dimensional orientation of grid cells within a neighbourhood. This method effectively captures variability in slope and aspect into a single measure^2^.

Bathymetry and rugosity are indicative of seabed morphology and have been used as predictors of species distribution and suitable habitat^3,4,5,6^. Rugosity values are scaled between 0 (no terrain variation) to 1 (complete terrain variation). Commonly, unconsolidated substrate, such as mud and sand, correspond to low rugosity values, thus, high rugosity values are associated to potential rocky substrate. This parameter is considered to be a useful surrogate for benthic diversity where there is no detailed information on sediment type and structure^7,8,9^. In order to have the same spatial resolution, all environmental data were aggregated at 0.25 x 0.25 degree using the ‘*raster*’ package^10^ in the R software^11^.

All environmental variables were explored for correlation, collinearity, outliers, and missing data before their use in the models following the approach of Zuur *et al.* (2010)^12^. SSS and distance to land were highly correlated (r = 0.81), and with a Generalized Variance Inflation Index (VIF) higher than 3. For this reason, separate model runs were performed including only one of each of the highly correlated variables (SSS or distance to land) to determine which one would explain more of the data variability.

The remaining potential source of variation on the juveniles’ dataset could be due to the individual behaviour of observers caused by random aspects, such as experience. Ignoring such non-independence of the data may lead to an invalid statistical inference. To remove any bias caused by observer-specific differences in the sampling, an observer effect was included as a random effect, because there was no interest in knowing the specific nature of the observer.


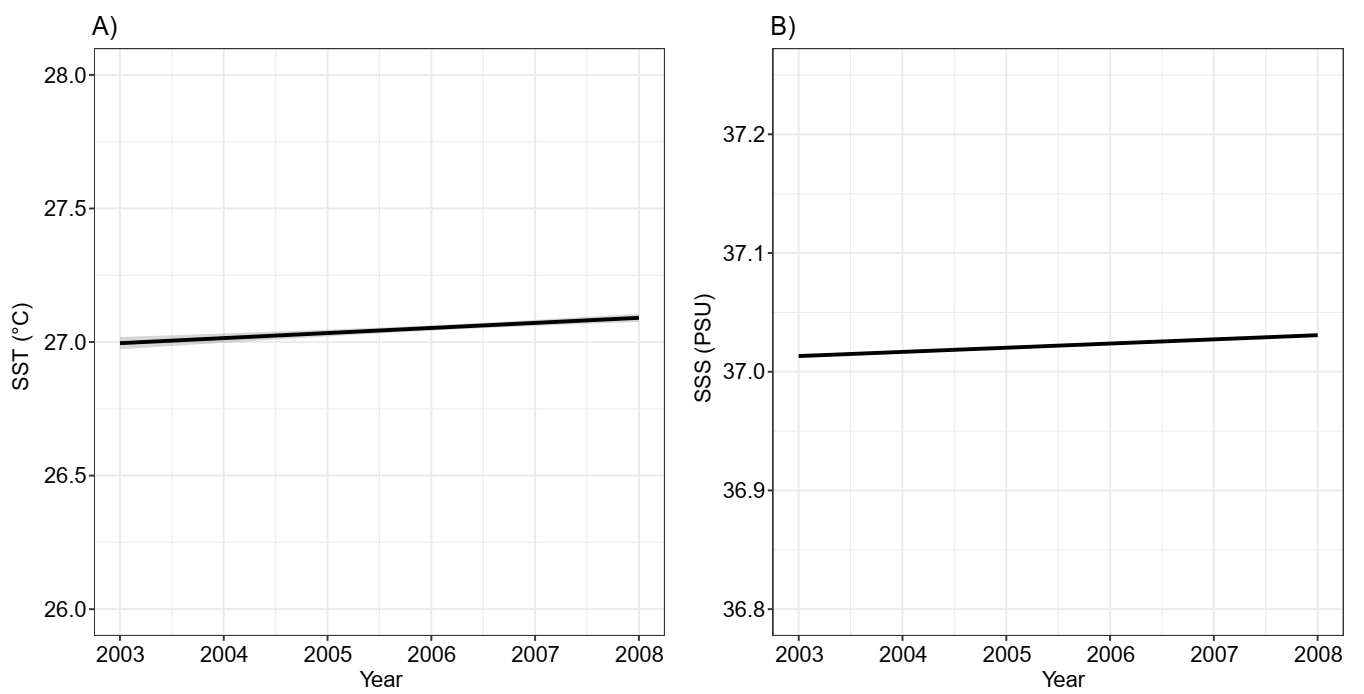


**Figure S1.** Temporal variation (2003-2008) in the Sea Surface Temperature (SST °C; A) and Sea Surface Salinity (SSS in PSU; B). Graphs were plotted using the package “ggplot2”^13^ in the R software^11^.


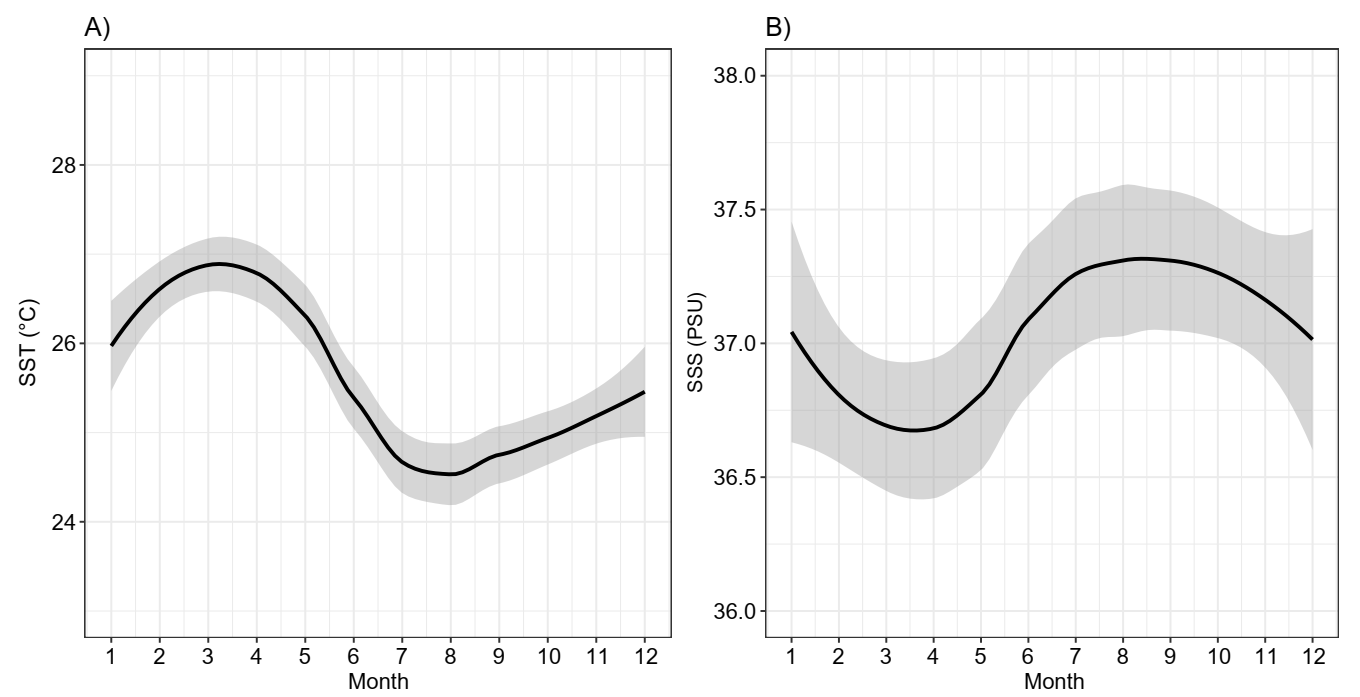


**Figure S2.** Annual variation (mean 2003-2008) in the Sea Surface Temperature (SST °C; A) and Sea Surface Salinity (SSS in PSU; B). Graphs were plotted using the package “ggplot2”^13^ in the R software^11^.

**Results**


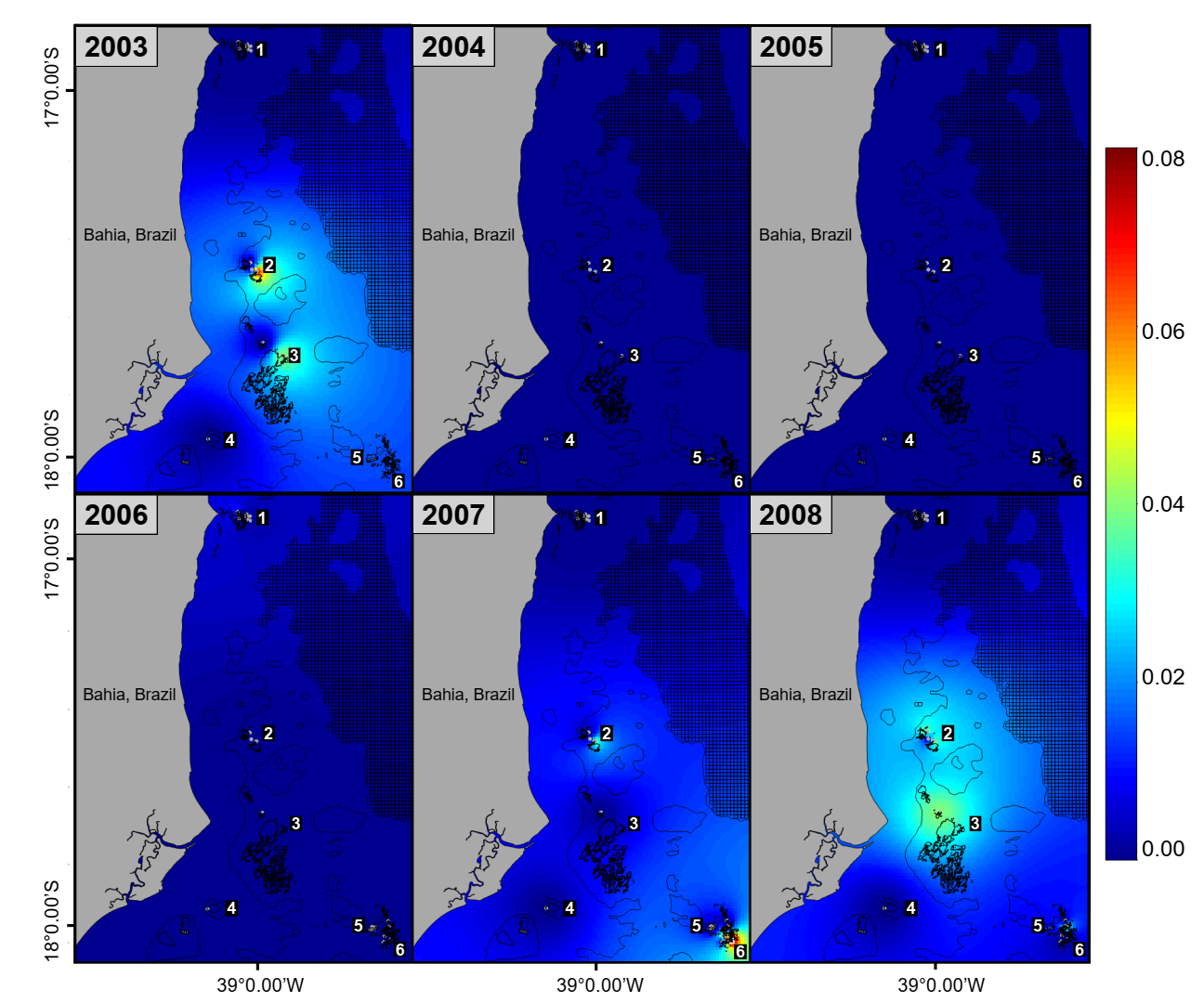


**Figure S3.** Predicted hotspots for *Scarus trispinosus*’ juveniles per year. 1 – Itacolomis reefs; 2 – Timbebas reefs; 3 – Parcel das Paredes reefs; 4 – Sebastião Gomes reefs; 5 – Abrolhos Archipelago; 6 – Parcel dos Abrolhos reefs. The maps were plotted using the packages “raster”^10^, “maptools”^14^ and “rworldmap”^15^ in the R software^11^.

**Table S2.** Model comparison of the 10 most relevant models for the *Scarus trispinosus*’ juveniles. Statistics acronyms are: WAIC = Watanabe-Akaike information criterion; RMSE = Root Mean Square Error; R^2^ = adjusted coefficient of determination. Predictors acronyms are: SST = Sea Surface Temperature; SSS = Sea Surface Salinity; DL = distance to land; R= rugosity; CCA = Calcareous Algae; Tu = Turf, FM= Fleshy macroalgae; SC = Stony coral; S = Sponge; Z = Zoanthids; FC= Fire coral; W = spatial random effect; T =temporal effect; O= observer effect. Relevant environmental predictors are indicated by *, *i.e.,* those predictors with 95% credibility intervals not covering zero. The best model is highlighted in bold.

| Model | WAIC | RMSE | R^2^ |
| --- | --- | --- | --- |
| **1 + SSS *+ R* + CCA* + Tu* + FM* + SC* + S* +FC *+Z*+W*+T*** | 1564 | 0.32 | 0.72 |
| 1 + SST + R* + CCA* + Tu* + FM* + SC* + S* +FC *+Z*+W*+T* | 1564 | 0.35 | 0.72 |
| 1 + DL* + R* + CCA* + Tu* + FM* + SC* + S* +FC *+Z*+W*+T* | 1583 | 0.38 | 0.68 |
| 1 + SSS* + R* + CCA* + Tu* + FM* + SC* + S* +FC *+Z*+W* | 1673 | 0.42 | 0.68 |
| 1 + SSS* + R* + CCA* + Tu* + FM* + SC* + S* +FC *+Z*+T* | 1698 | 0.43 | 0.60 |
| 1 + SSS* + R* + CCA* + Tu* + FM* + SC* + S* +FC *+Z* | 1878 | 0.45 | 0.58 |
| 1 + SSS* + R* + CCA* + Tu* + FM* + SC* + S* +FC *+Z*+ O | 1879 | 0.45 | 0.58 |
| 1 + SSS* + CCA* + Tu* + FM* + SC* + S* +FC *+Z*+W*+T* | 1585 | 0.36 | 0.69 |
| 1 + SSS* + R* + W*+T* | 1598 | 0.36 | 0.56 |
| 1 + SSS* + W*+T* | 1585 | 0.36 | 0.55 |

**Table S3.** Model comparison of the most relevant models for *Scarus trispinosus*’ abundance trends. Statistics acronyms are: WAIC = Watanabe-Akaike information criterion; CPO = Conditional Predictive Ordinates; AR1 = autoregressive model of order 1; RW1 = random walk model order 1; RW2 = random walk model order 2. The best model is highlighted in bold. *Most parsimonious model.

| Juveniles | Model | WAIC | CPO | Adults | Model | WAIC | CPO |
| --- | --- | --- | --- | --- | --- | --- | --- |
| Sites combined | AR1 | 73.74 | 34.22 | Sites combined | **AR1** | **59.13** | **36.87** |
|  | RW1 | 71 | 33.98 |  | RW1 | 59.29 | 36.03 |
|  | **RW2** | **60.81** | **31.16** |  | RW2 | 60.22 | 38.05 |
| Itacolomis reefs | AR1 | 38.28 | 32.57 | Itacolomis reefs | **AR1** | **50.74** | **31.72** |
|  | **RW1** | **36.93** | **33.38** |  | RW1 | 51.08 | 31.78 |
|  | RW2 | 69.04 | 43.71 |  | RW2 | 56.45 | 35.14 |
| Timbebas reefs | AR1 | 50.01 | 25.12 | Timbebas reefs | **AR1** | **50.05** | **33.00** |
|  | RW1 | 49.62 | 24.91 |  | RW1 | 50.86 | 34.45 |
|  | **RW2** | **46.23** | **23.32** |  | RW2 | 50.83 | 36.27 |
| Parcel das Paredes reefs | AR1 | 57.03 | 28.91 | Parcel das Paredes reefs | **AR1** | **50.48** | **32.70** |
|  | RW1 | 56.93 | 28.74 |  | RW1 | 50.60 | 32.51 |
|  | **RW2** | **47.96** | **24.70** |  | RW2 | 51.43 | 33.91 |
| Abrolhos Archipelago | AR1 | 11 | 5.54 | Abrolhos Archipelago | AR1 | 24.44 | 15.94 |
|  | RW1 | 11 | 5.54 |  | **RW1** | **24.42** | **15.83** |
|  | **RW2*** | **11.96** | **12.19** |  | RW2 | 45.99 | 30.46 |
| Parcel dos Abrolhos reefs | AR1 | 29.51 | 15.49 | Parcel dos Abrolhos reefs | AR1 | 35.97 | 18.54 |
|  | RW1 | 29.52 | 15.37 |  | RW1 | 35.93 | 18.47 |
|  | **RW2** | **19.40** | **14.55** |  | **RW2** | **31.24** | **19.24** |

**References**

1. Sbrocco, E. & Barber, P. MARSPEC: Ocean climate layers for marine spatial ecology. *Ecology*, **94**(4), 979–979 (2013).
2. Sappington, J. M., Longshore, K. M. & Thompson, D. B. Quantifying Landscape Ruggedness for Animal Habitat Analysis: A Case Study Using Bighorn Sheep in the Mojave Desert. *J. Wildlife Manage.* **71,** 1419–1426 (2007).
3. Gratwicke, B. & Speight, M. R. The relationship between fish species richness, abundance and habitat complexity in a range of shallow tropical marine habitats. *J. Fish Biol.* **66**, 650–667 (2005).
4. Pittman, S. J. & Brown, K. A. Multi-scale approach for predicting fish species distributions across coral reef seascapes. *PLoSOne* **6,** e20583 (2011).
5. Lauria, V., Gristina, M., Attrill, M. J., Fiorentino, F. & Garofalo, G. Predictive habitat suitability models to aid conservation of elasmobranch diversity in the central Mediterranean Sea. *Sci. Rep.* **5,** 13245 (2015).
6. Lauria, V., Power, A., Lordan, C., Weetman, A. & Johnson, M. P. Spatial transferability of habitat suitability models of Nephrops norvegicus among fished areas in the Northeast Atlantic: sufficiently stable for marine resource conservation? *PLoSOne*, **10,** e0117006 (2015).
7. Pittman, S. J., Christensen, J. D., Caldow, C., Menza, C. & Monaco, M. E. Predictive mapping of fish species richness across shallow-water seascapes in the Caribbean. *Ecol. Model.* **204,** 9–21 (2007).
8. Dunn, D. C. & Halpin, P. N. Rugosity-based regional modeling of hard-bottom habitat. *Mar. Ecol. Prog. Ser.* **377,** 1–11 (2009).
9. McArthur, M. A. *et al.* On the use of abiotic surrogates to describe marine benthic biodiversity. *Estuar. Coast. Shelf*. **88,** 21–32 (2010).
10. Hijmans, R. J. raster: Geographic Data Analysis and Modeling. R package version 3.0-7. https://CRAN.R-project.org/package=raster (2019).
11. R: A Language and Environment for Statistical Computing. R Core Team. R Foundation for Statistical Computing. Vienna, Austria. Available at https://www.R-project.org. (2020).
12. Zuur, A. F., Ieno, E. N. & Elphick, C. S. A protocol for data exploration to avoid common statistical problems. *Methods Ecol. Evol.* **1,** 3–14 (2010).
13. Wickham H. ggplot2: Elegant Graphics for Data Analysis. Springer-Verlag New York. ISBN 978-3-319-24277-4. Available at https://ggplot2.tidyverse.org. (2016).

14. Bivand, R. & Lewin-Koh, N. maptools: Tools for Handling Spatial Objects. R package version 1.0-1. Available at https://CRAN.R-project.org/package=maptools (2020).

15. South, A. rworldmap: A New R package for Mapping Global Data. The R Journal 3(1): 35–43. Available at http://journal.r-project.org/archive/2011-1/RJournal_2011-1_South.pdf. (2011).
